# Arginyl dipeptides increase the frequency of NaCl-elicited responses via epithelial sodium channel alpha and delta subunits in cultured human fungiform taste papillae cells

**DOI:** 10.1038/s41598-017-07756-x

**Published:** 2017-08-08

**Authors:** Jiao-Jiao Xu, Nadia Elkaddi, Alvaro Garcia-Blanco, Andrew I. Spielman, Alexander A. Bachmanov, Hau Yin Chung, Mehmet Hakan Ozdener

**Affiliations:** 1 Food and Nutritional Sciences Programme, School of Life Sciences, The Chinese University of Hong Kong, Shatin, N.T. Hong Kong SAR, China; 2 Monell Chemical Senses Center, Philadelphia, PA 19104 USA; 3 College of Dentistry, New York University, New York, NY 10010 USA

## Abstract

Salty taste is one of the five basic tastes and is often elicited by NaCl. Because excess sodium intake is associated with many health problems, it could be useful to have salt taste enhancers that are not sodium based. In this study, the regulation of NaCl-induced responses was investigated in cultured human fungiform taste papillae (HBO) cells with five arginyl dipeptides: Ala-Arg (AR), Arg-Ala (RA), Arg-Pro (RP), Arg-Glu (RE), and Glu-Arg (ER); and two non-arginyl dipeptides: Asp-Asp (DD) and Glu-Asp (ED). AR, RA, and RP significantly increased the number of cell responses to NaCl, whereas no effect was observed with RE, ER, DD, or ED. We also found no effects with alanine, arginine, or a mixture of both amino acids. Pharmacological studies showed that AR significantly increased responses of amiloride-sensitive but not amiloride-insensitive cells. In studies using small interfering RNAs (siRNAs), responses to AR were significantly decreased in cells transfected with siRNAs against epithelial sodium channel ENaCα or ENaCδ compared to untransfected cells. AR dramatically increased NaCl-elicited responses in cells transfected with NHE1 siRNA but not in those transfected with ENaCα or ENaCδ siRNAs. Altogether, AR increased responses of amiloride-sensitive cells required ENaCα and ENaCδ.

## Introduction

Humans perceive five basic tastes – bitter, sweet, umami, sour, and salty – via taste receptor cells clustering in the taste buds of specialized papillae in the oral cavity^1, 2^. Taste papillae are divided into three morphological types, fungiform, circumvallate, and foliate papillae, which are located on the anterior, posterior, and lateral sides of the tongue, respectively^3^. Each taste bud contains at least four types of cells: types I–IV^4^. Type I cells express glutamate-aspartate transporters (GLAST) for glutamate. They also express NTPDase2, a plasma-membrane-bound nucleotidase involved in extracellular ATP hydrolysis, and ROMK, a potassium channel that may be responsible for maintaining K^+^ homeostasis. Type II cells express all the elements of the taste transduction cascade for sweet, bitter, and umami taste. Unlike type I and type II cells, type III cells express synaptic membrane proteins, neural cell adhesion molecule (NCAM), and synaptosomal-associated protein 25 (SNAP-25). Type IV cells are proliferative cells located at the bottom of the taste bud^4, 5^. Taste plays a large role what we choose to eat, and there is a strong correlation between consumption of high-salt food and many health problems^1, 6–8^. Currently, daily individual sodium consumption in most countries is reported to be more than twice the amount recommended by the World Health Organization^9^. Much effort has been made to decrease sodium consumption, but salt substitution has been limited mainly to infant formulas and baked foods^10, 11^. As yet, no compounds are available that can effectively substitute for the taste of sodium chloride in food. Therefore, it is imperative to search for a salty taste enhancer as an alternative approach to reduce sodium consumption in the general population.

Salty taste is recognized by salt receptors in the oral cavity, and evidence indicates that epithelium sodium channel (ENaC) subunits may play roles in this recognition and that at least two pathways, amiloride-sensitive and amiloride-insensitive, are involved in salty taste transduction^12, 13^. Amiloride and its derivative benzamide are high-affinity blockers of ENaC^6, 7^. In rodents, approximately 65% of fungiform papillae taste cells exhibit functional amiloride-sensitive Na^+^ currents, whereas only 35% of foliate papillae cells are amiloride-sensitive. In contrast, taste cells of the circumvallate papillae are completely insensitive to amiloride, although ENaCα mRNA and immunoreactivity to the purified amiloride-sensitive Na^+^ channel proteins have been detected in those cells^3^. The amiloride-sensitive pathway is Na^+^ specific and mediated by taste receptor cells expressing ENaC, a member of the degenerin/epithelial sodium channel (DEG/ENaC) family of non-voltage-gated ion channels^1, 14, 15^. However, the amiloride-insensitive pathway is cation nonselective, recognizing Na^+^, K^+^, and NH_4_^+^ salts^12, 16^.

Amino acids interact with many receptors; the tastes of individual amino acids are complex and in human sensory studies are described by more than one taste characteristic^17, 18^. Much less is known about the tastes of dipeptides, made of two amino acids joined by a planar peptide linkage, and there is no strict relationship between the taste of dipeptides and the constituent amino acids^19, 20^. Previous reports indicate that arginine amino acid and the arginyl dipeptides Ala-Arg (AR), Arg-Ala (RA), and Arg-Pro (RP) may enhance salty taste, increasing the salty taste of 50 mM NaCl in both aqueous and model broth solutions in human sensory evaluations^9^. However, the underlying cellular mechanism is not known.

In this study, we used cultured human taste cells to explore the mechanisms underlying the previously reported enhancement of salty taste by alanyl-arginine peptides. We examined effects on cellular responses to NaCl elicited by five arginyl dipeptides: AR, RA, RP, Arg-Glu (RE), and Glu-Arg (ER); and two non-arginyl dipeptides: Asp-Asp (DD) and Glu-Asp (ED). We found that the AR arginyl dipeptide increased the number of NaCl-induced responses, acting on amiloride-sensitive cells, targeting ENaCα and ENaCδ receptors. This work provides mechanistic information on the enhancement of NaCl-elicited responses by dipeptides and thus may suggest some alternatives to reduce sodium consumption.

## Results

### Effect of dipeptides on cultured human fungiform papillae cells

**Figure 1:**
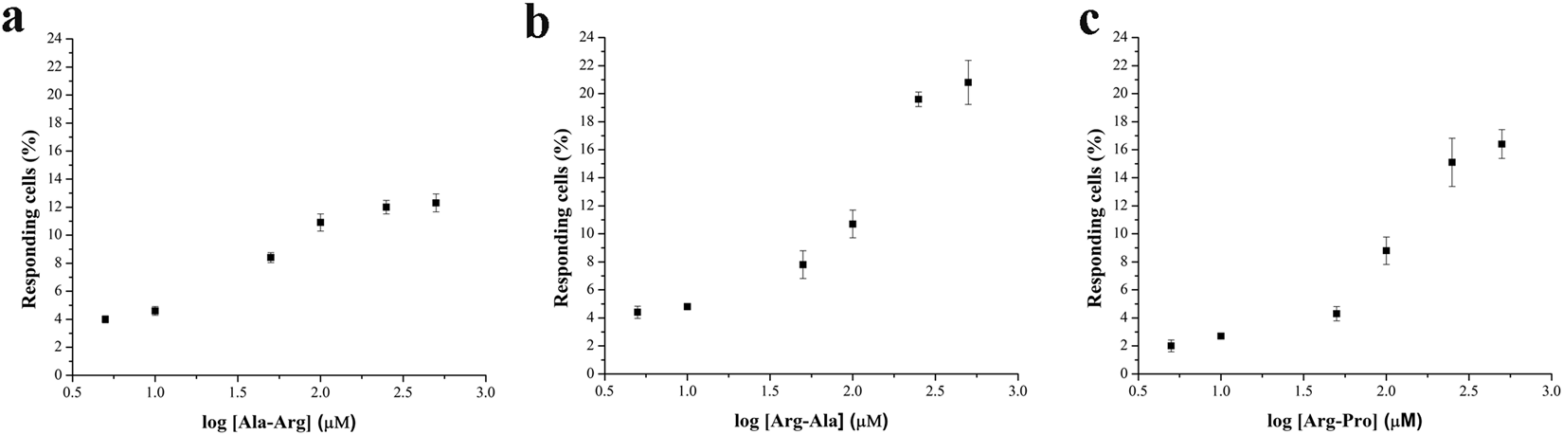
Arginyl dipeptides Ala-Arg (**a**), Arg-Ala (**b**), and Arg-Pro (**c**) elicited responses in a concentration-dependent manner. HBO cells were stimulated with six different dipeptide concentrations: 5, 10, 50, 100, 250, and 500 µM (x-axis) and each point represents the average of data collected from independent experiments. The percentage of cells responding were measured by intracellular Ca^2+^ changes (y-axis). Each experiment was performed eight times. For each panel the peak number of responding cells/total cells examined were: 184/582 (**a**), 92/276 (**b**) and 110/430 (**c**).

We evaluated Ca^2+^ responses elicited by dipeptides in cultured human fungiform taste (HBO) cells. Dipeptides at different concentrations (5, 10, 50, 100, 250, and 500 µM) elicited a concentration-dependent response relationship, with EC_50_ values of 45, 122, 106, 89, 114, 100, and 82 µM for AR, RA, RP, RE, ER, DD, and ED, respectively (Fig. 1, Fig. S1). In addition, the minimum and maximum effective concentrations of AR were 10 and 100 μM, respectively (Fig. 1a). AR at a concentration of 50 µM was close to the EC_50_ value. Therefore, in subsequent experiments, AR and RA were used at concentrations of 10, 50, and 100 μM. Similarly, other arginyl dipeptides and non-arginyl dipeptides at concentrations of 50 and 100 μM were used for further investigation.

### Dipeptides increase the percentage of cells responding to NaCl

**Figure 2:**
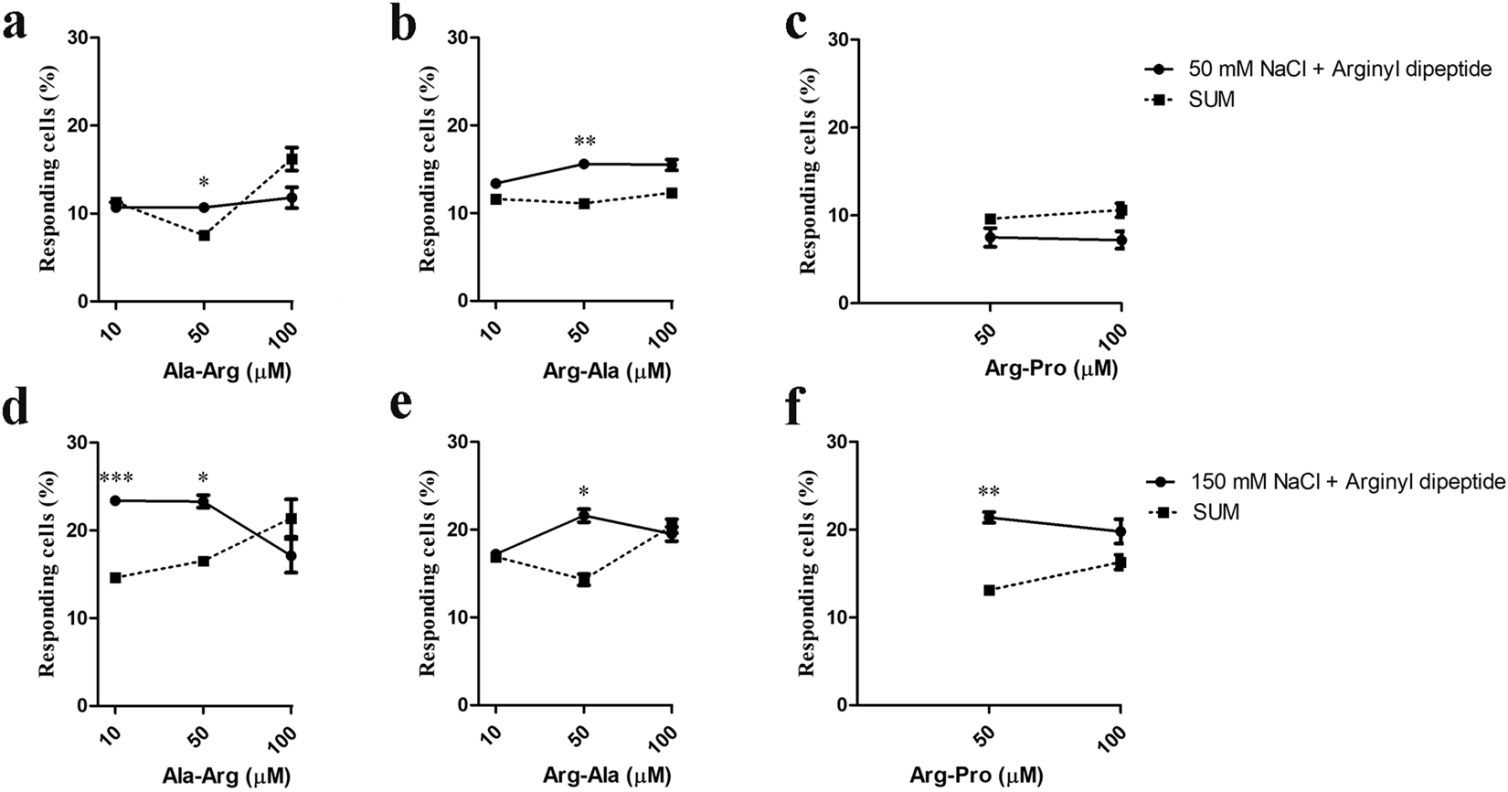
Effects of Ala-Arg (AR), Arg-Ala (RA), and Arg-Pro (RP) on NaCl-elicited responses. HBO cells were consecutively stimulated by dipeptide alone, NaCl alone (50 mM, (**a–c**); 150 mM, (**d–f**)), and a mixture of dipeptide and NaCl. SUM is the mathematical addition of individual responses (NaCl alone + dipeptide alone). The percentage of responding cells was determined by measuring intracellular Ca^2+^ changes. Each experiment was performed at least nine times. *p < 0.05, **p < 0.01, ***p < 0.001, comparing the mixture (dipeptide and NaCl) and SUM. For each panel the peak number of responding cells/total cells examined were: 91/1990 (**a**), 84/1706 (**b**), 20/394 (**c**), 174/1640 (**d**) 188/1931 (**e**), and 110/1143 (**f**).

To investigate if dipeptides could alter the number of responses induced by NaCl, HBO cells were consecutively stimulated with three different stimuli: dipeptide alone, NaCl alone, and a mixture of dipeptide and NaCl. At least a 5-min washout between stimulations ensured all cells returned to their baseline condition. As shown in Fig. 2d, 10 μM AR significantly increased the percent of cell responding to 150 mM NaCl, with 23.4% of cells (144 of 616 cells, n = 11) responding to the mixture compared to a summed individual response of 14.6% – an increase of 60.0%. While 10 μM AR did not alter the frequency of responses to 50 mM NaCl (Fig. 2a), 50 μM AR influenced the number of responding cells, with an increase from 7.5% to 10.7% (84 of 787 cells, n = 17) with 50 mM NaCl and from 16.5% to 23.3% (158 of 679 cells, n = 13) with 150 mM NaCl (Fig. 2a,d). However, these results should be interpreted with caution because the summated responses to 50 mM NaCl plus AR did not increase monotonically. Responses to 50 or 150 mM NaCl were not affected by 100 μM AR (Fig. 2a,d). The representative *F*_340_/*F*_380_ ratiometric images of fura-2AM-loaded HBO cells are shown in Fig. S2.

Similar results were observed in the presence of the dipeptide RA (Fig. 2b,e). RA at 50 μM significantly increased the percent of cells responding to 50 and 150 mM NaCl from 11.1% to 15.6% (80 of 514 cells, n = 9) and from 14.3% to 21.6% (124 of 574 cells, n = 10), respectively; these represent increases of 40.4% and 51.2%, respectively. However, responses to 50 or 150 mM NaCl were not increased when presented with 10 or 100 μM RA (Fig. 2b,e). Again, caution is warranted because the sums did not increase monotonically.

At 50 μM RP, the percent responding cells significantly increased for 150 mM NaCl by 38.6%, (189 of 885 cells, n = 19) in the mixture. However, no effect on response frequency was observed at 50 mM NaCl. Neither 50 nor 150 mM NaCl-elicited responses were increased by 100 μM RP (Fig. 2c,f). Also, both arginyl dipeptides (RE and ER) and non-arginyl dipeptides (DD and ED) at 50 and 100 μM did not affect the number of cell responses at 50 and 150 mM NaCl (Fig. S3a,b).

Finally, alanine (Ala) and arginine (Arg), the component amino acids of the most active dipeptide AR, were further investigated alone and as a mixture for potential effects. Neither alanine nor arginine alone at concentrations of 10, 50, and 100 μM increased cell responses when mixed with NaCl (50 and 150 mM; Fig. S4a), nor did mixtures of alanine (25 and 50 μM) and arginine (25 and 50 μM; Fig. S4b).

### The breadth of tuning of AR with other basic taste stimuli in HBO cells

**Figure 3:**
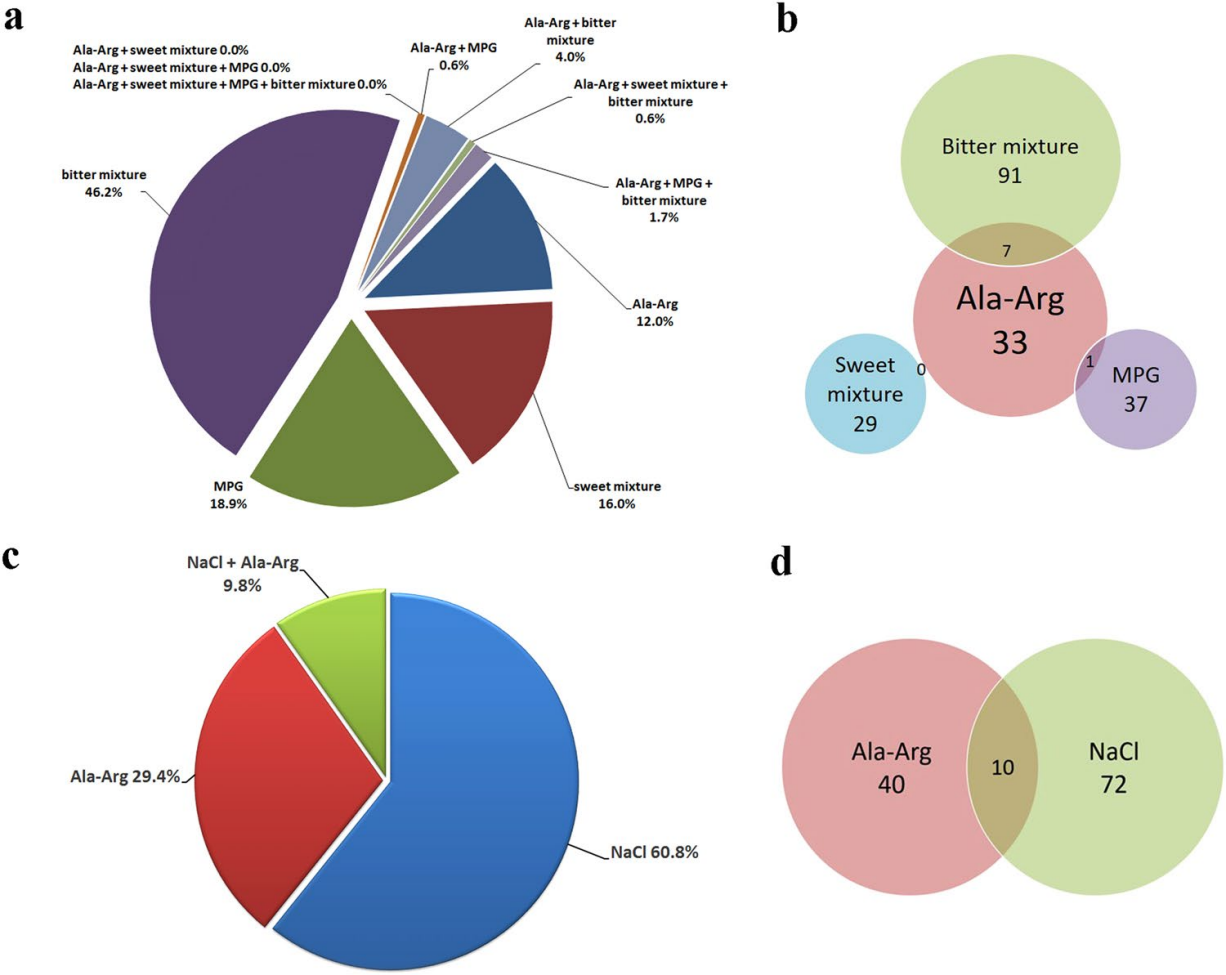
The breadth of tuning of Ala-Arg (AR) with sweet, bitter, umami, and salty taste. Sweet mixture (100 mM glucose and 1 mM sucralose), bitter mixture (2 mM phenylthiocarbamide, 2 mM denatonium benzoate, and 5 mM salicin), 5 mM monopotassium glutamate (MPG), and 150 mM NaCl were used as sweet, bitter, umami, and salt stimuli, respectively. **(a)** HBO cells were consecutively stimulated with four different stimuli: AR, sweet mixture, bitter mixture, and MPG. Of 576 HBO cells, 175 responded to at least one stimulus, as measured by Ca^2+^ response. The experiment was performed eleven times. **(b)** Among 33 AR-responding cells, 1 and 7 cells responded to MPG and bitter mixture, respectively, but no AR-responding cells responded to the sweet mixture. **(c)** HBO cells were consecutively stimulated with NaCl (150 mM) and AR (50 µM). The experiment was performed thirteen times. At least one stimulus elicited a Ca^2+^ response in 102 of 679 HBO cells. **(d)** Among 40 AR-responding cells, 10 cells responded to NaCl.

AR exhibited the most consistent effect on NaCl response among the seven dipeptides, AR, RA, RP, RE, ER, DD, and ED. Therefore, we further investigated how AR affected the number of NaCl-responding cells. The breadth of tuning of AR at a concentration of 50 μM with other basic tastes was evaluated by calcium imaging. HBO cells were used to evaluate their responses to four different stimuli: AR, monopotassium glutamate (MPG), sweet mixture, and bitter mixture. The stimuli were applied sequentially but in random order. Responses to basic taste stimuli and to AR are shown in Fig. 3. Responses of each cell to a single stimulus or multiple stimuli were recorded, and 175 responding cells (30.4%, n = 11) were identified among the total of 576 taste cells examined. Among the 175 responding cells, 21 (12.0%), 28 (16.0%), 33 (18.9%), and 81 (46.2%) responded to AR, sweet mixture, MPG, and bitter mixture alone, respectively; 1 (0.6%), 7 (4.0%), 1 (0.6%), and 3 (1.7%) cells responded to both AR and MPG; AR and bitter mixture; AR, sweet, and bitter mixtures; and AR, MPG, and bitter mixture, respectively (Fig. 3a). Among the 33 AR-responding cells, 1 (3.0%) and 7 (21.2%) responded to MPG, and bitter mixture, respectively, but none responded to the sweet mixture (Fig. 3b).

The breadth of tuning of cells responding to AR and NaCl was also investigated. At least one stimulus elicited a response in 102 of 679 (15.0%, n = 13) taste cells. Of these 102 responding cells, 30 (29.4%), 62 (60.8%), and 10 (9.8%), respectively, responded to 50 μM AR alone, 150 mM NaCl alone, and both AR and NaCl (Fig. 3c). Of the 40 AR-responding cells, 10 (25%) also responded to NaCl (Fig. 3d).

In summary, the sensory response profile of AR-responsive cells was distinct from those that responded to umami, sweet, bitter, and salty stimuli.

### Signal transduction pathway utilized by the AR dipeptide

To investigate which receptor interacts with AR, we recorded HBO cell responses to AR with the specific blockers amiloride (ENaC blocker), cariporide (solute carrier family 9, subfamily A, member 1 [NHE1] blocker), Ki16425 (lysophosphatidic acid receptor 1 [LPAR1] blocker), NPS2143 (calcium sensing receptor [CaSR] blocker), and U73122 (phospholipase C beta 2 [PLCβ2] blocker).

**Figure 4:**
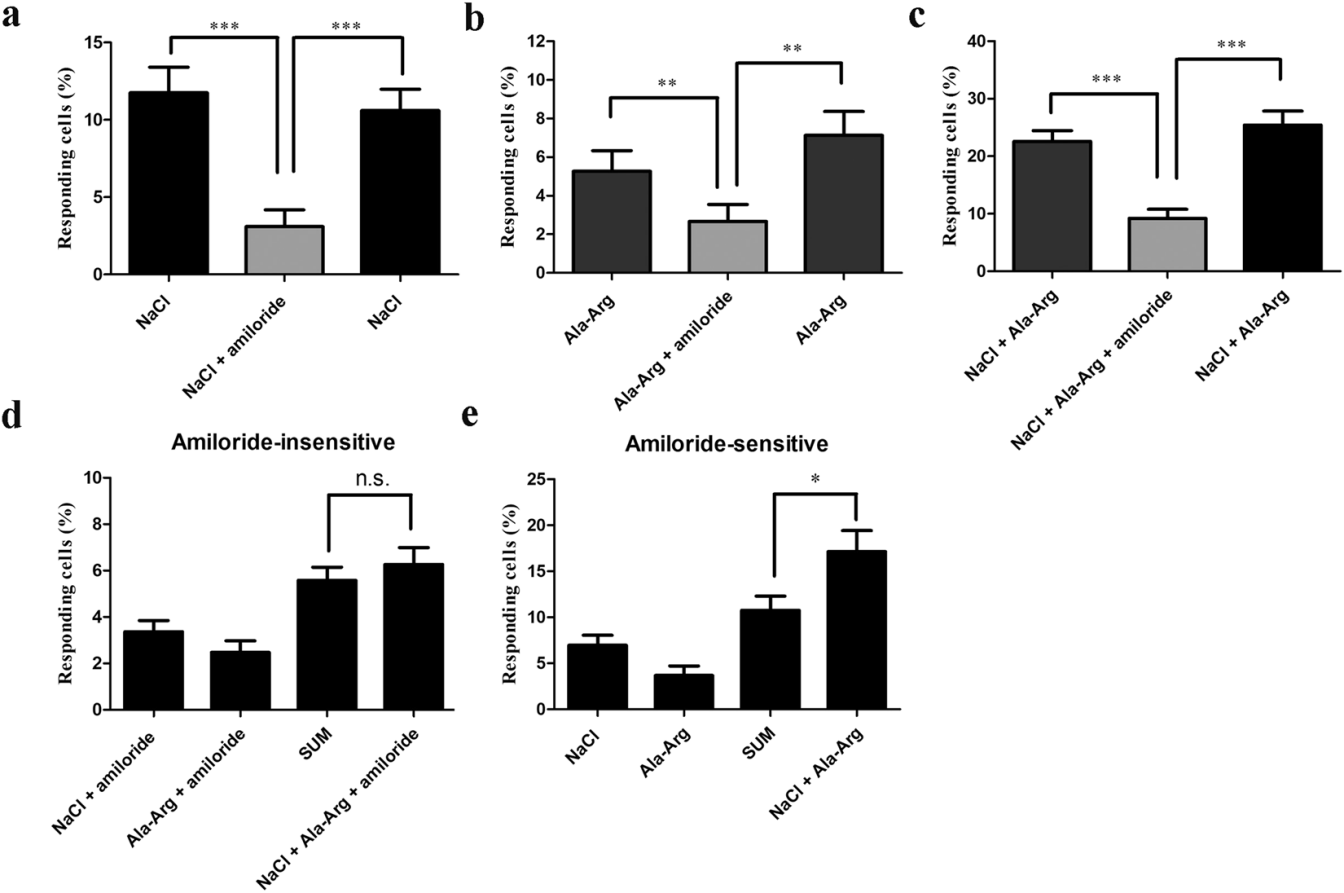
Signaling pathway utilized by Ala-Arg (AR). **(a**–**c)** Amiloride (50 μM) significantly suppressed responses induced by NaCl **(a)**, AR **(b)**, and the mixture of NaCl and AR **(c)**. HBO cells were stimulated with three stimuli; the third stimulus was the same as the first one to demonstrate that cells were the responsive equally first stimulus. Each experiment was performed six times. **(d** and **e)** AR (50 μM) increased the percent cell responses to 150 mM NaCl via the amiloride-sensitive but not the amiloride-insensitive pathway. Each experiment was performed nineteen times. The concentration of amiloride was 50 μM. The percentage of responding cells was determined by intracellular Ca^2+^ changes. *p < 0.05, **p < 0.01, ***p < 0.001, n.s., not significant.

As a control, amiloride significantly inhibited the number of HBO cells that responded to 150 mM NaCl. The number of NaCl-responding cells were reduced by 70.5%, from 11.2% (48 of 430 cells, n = 10) to 3.3% (14 of 430 cells; Fig. 4a). Amiloride also significantly suppressed the number of 50 µM AR-responding cells by 52.4%, from 5.3% (21 of 397 cells, n = 9) to 2.5% (10 of 397 cells, n = 9). The responding cells increased again when stimulated with AR at the end of the stimulus test (Fig. 4b). Similarly, amiloride dramatically reduced the number of cells responding to the mixture of NaCl and AR: the responses were reduced by 59.6%, from 22.5% (55 of 244 cells, n = 6) to 9.1% (36 of 396 cells, n = 10), but the responding cells again increased to 24.0% (95 of 396 cells, n = 10) when stimulated by the mixture of NaCl and AR at the end of the stimulus test (Fig. 4c). Nevertheless, amiloride did not completely block the cell activity in the presence of either AR or the mixture of AR + NaCl (Fig. 4b,c). Taken together, these results indicate that AR increased the number of NaCl-elicited responses mostly via amiloride-sensitive pathways. However, one or more amiloride-insensitive pathways are likely to be involved given that amiloride did not completely inhibit the AR + NaCl responses.

We examined the effect of AR on NaCl responses of amiloride-insensitive cells. Mixtures of NaCl and amiloride, and AR and amiloride were responded to by 3.3% (38 of 1139 cells, n = 28) and 2.4% (22 of 908 cells, n = 19) of total taste cells, respectively. There was no significant difference in numbers of responding cells between the mixture of NaCl, AR, and amiloride (6.3%, 79 of 1,254 cells, n = 32) and the the calculated sum of responses to the individual compounds (5.7%; Fig. 4d). Although AR did not increase responses of amiloride-insensitive cells, there was a large increase in the absence of amiloride when comparing the numbers of cells responding to the mixture of NaCl and AR (17.0%) compared to the calculated sum of cells responding to each compound (10.8%; Fig. 4e). In sum, AR increased NaCl-induced responses among amiloride-sensitive cells but not among amiloride-insensitive cells.

Similar experiments were carried out with other specific inhibitors. Cariporide did not inhibit AR-induced responses, with 6.3% (14 of 221 cells, n = 4) of cells responding to AR alone or the mixture of AR and cariporide (Fig. S5a). Ki16425 also did not suppress AR-elicited responses, with 4.2% (8 of 191 cells, n = 4) of cells responding to either AR alone or the mixture of AR and Ki16425 (Fig. S5b). NPS2143 exhibited no inhibition, with 5.3% (7 of 133 cells, n = 3) of cells responding to AR alone or the mixture of AR and NPS2143 (Fig. S5c). Similarly, no significant difference in response was observed between AR alone (4.0%, 4 of 100 cells) and AR plus U73122 (6.0%, 6 of 100 cells, n = 2; Fig. S5d). That none of these inhibitors suppressed the AR-elicited responses indicates that AR’s activation of HBO cells is not via NHE1, LPAR1, CaSR, PLCβ2 or signaling pathways depending on these proteins.

### Taste cell responses to NaCl increased by AR depend on ENaCα and ENaCδ subunits

Amiloride significantly inhibited the number of cells that responded to either AR or the mixture of NaCl and AR. To confirm that AR increased the number of NaCl-elicited responses via ENaC and to investigate which subunit(s) of ENaC were involved, ENaCα and ENaCδ were downregulated using small interfering RNAs (siRNAs). siRNA knockdown of NHE1 was used as a negative control.

**Figure 5:**
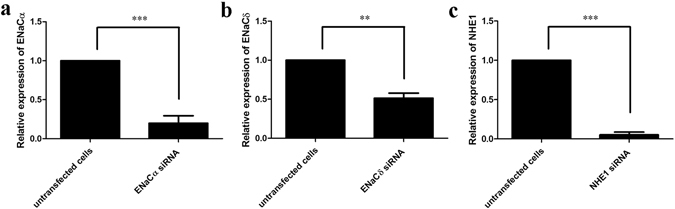
ENaCα, ENaCδ, and NHE1 mRNA expression in both transfected and untransfected cells. mRNA expressions of ENaCα **(a)**, ENaCδ **(b)**, and NHE1 **(c)** were significantly decreased in siRNA-transfected cells. Each experiment was performed three times. *p < 0.05, **p < 0.01, ***p < 0.001, transfected vs. untransfected cells.

**Figure 6:**
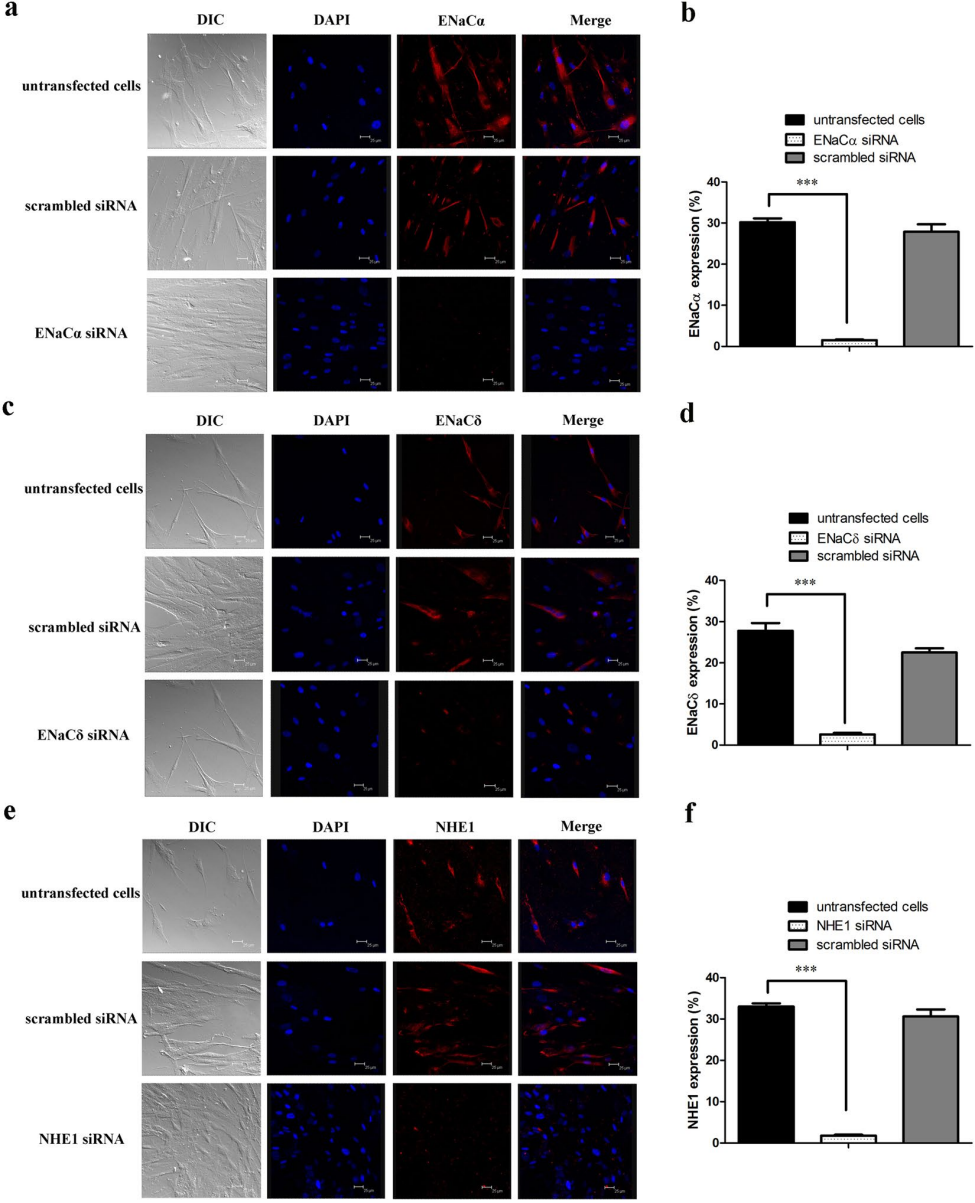
ENaCα, ENaCδ, and NHE1 protein expression in transfected and untransfected cells. ENaCα, ENaCδ, and NHE1 are visualized with a red color; nuclei are stained with a blue color. **(a)** HBO cells express ENaCα proteins. **(b)** Percent cells expressing ENaCα in untransfected cells (30.2%, 88 of 291 cells), ENaCα siRNA transfected cells (1.5%, 5 of 327 cells), and scrambled siRNA- transfected cells (27.9%, 69 of 247 cells). **(c)** The ENaCδ proteins express in HBO cells. **(d)** Percent cells expressing ENaCδ in untransfected cells (27.8%, 49 of 176 cells), ENaCδ siRNA transfected cells (2.6%, 6 of 232 cells), and scrambled siRNA-transfected cells (22.5%, 29 of 129 cells). **(e)** Immunoreactivity of NHE1 antibodies demonstrates the presence of NHE1 expression in HBO cells. **(f)** Percent cells expressing NHE1 in untransfected cells (33.0%, 69 of 209 cells), NHE1 siRNA-transfected cells (1.8%, 5 of 280 cells), and scrambled siRNA-transfected cells (30.6%, 19 of 62 cells). Each experiment was performed three times. *p < 0.05, **p < 0.01, ***p < 0.001.

Quantitative real-time RT-PCR (qPCR) and immunocytochemistry were used to verify siRNA knockdown of gene and protein expression, respectively. qPCR indicated that ENaCα, ENaCδ, and NHE1 mRNA expression was reduced by siRNA by 80.1%, 48.7%, and 94.8%, respectively (Fig. 5a–c). Immunocytochemistry demonstrated that the number of cells expressing ENaCα, ENaCδ, and NHE1 decreased when treated with siRNAs from 30.2% (88 of 291 cells) to 1.5% (5 of 327 cells), from 27.8% (49 of 176 cells) to 2.6% (6 of 232 cells), and from 33.0% (69 of 209 cells) to 1.8% (5 of 280 cells), respectively (Fig. 6b,d,f). No differences in expression were observed in untransfected cells or cells that received scrambled siRNAs (Fig. 6a–f).

**Figure 7:**
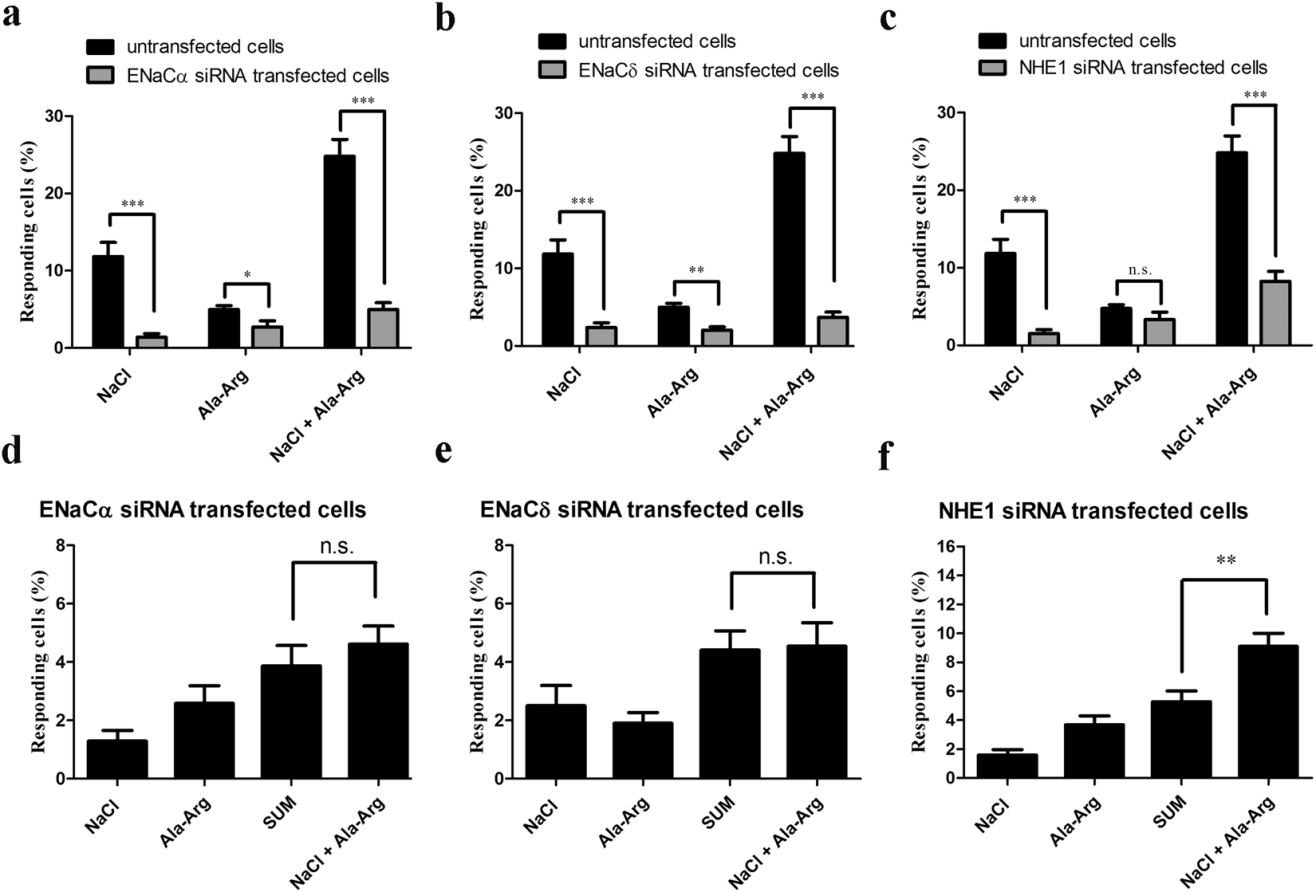
Signaling pathways of Ala-Arg (AR) in siRNA study. **(a**–**c)** NaCl (150 mM), AR (50 µM), and the mixture of NaCl and AR elicited responses in both siRNA-transfected and untransfected cells. Each experiment was performed eleven times. **(d**–**f)** AR regulation of cellular responses to NaCl in siRNA-transfected cells. Each experiment was performed seventeen times. Cell responses were measured by intracellular Ca^2+^ changes. *p < 0.05, **p < 0.01, ***p < 0.001, n.s., not significant.

To determine which ENaC subunit(s) might interact with AR, we tested the responses of siRNA-transfected cells. Treatment with siRNAs against ENaCα, ENaCδ, and NHE1 significantly decreased the numbers of taste cells responding to 150 mM NaCl alone from baseline of 11.7% (95 of 811 cells, n = 15) to 1.5% (8 of 545 cells, n = 12), 2.3% (13 of 574 cells, n = 11), and 1.3% (7 of 523 cells, n = 11), respectively (Fig. 7a–c). siRNAs against ENaCα and ENaCδ reduced the number of cells responding to AR alone from baseline of 4.8% (39 of 811 cells, n = 15) to 2.6% (14 of 545 cells, n = 12) and 2.1% (12 of 574 cells, n = 11), respectively (Fig. 7a,b). No significant differences in AR-responsive cells were observed between untransfected (4.8%, 39 of 811 cells, n = 15) and NHE1 siRNA-transfected cells (3.1%, 16 of 523 cells, n = 11; Fig. 7c). These results may suggest that the AR-induced responses in HBO cells act through ENaCα and ENaCδ receptors but not the NHE1 receptor.

Responses of taste cells to the mixture of NaCl and AR were reduced from 24.8% (201 of 811 cells, n = 15) to 5.1% (28 of 545 cells, n = 12), 3.8% (22 of 574 cells, n = 11), and 8.0% (42 of 523 cells, n = 11), by siRNAs to ENaCα, ENaCδ, or NHE1 was respectively (Fig. 7a–c). No significant differences were observed between untransfected and scrambled siRNA-transfected cells when stimulated with NaCl, AR, and the mixture of NaCl and AR (data not shown). ENaCα and ENaCδ siRNA markedly decreased the number of cells responding to the mixture of NaCl and AR. However, NHE1 siRNA decreased only the number of cells responding to the NaCl, not AR.

ENaCα siRNA did not alter the number of cells responding to the NaCl and AR mixture (4.4%, 37 of 843 cells, n = 18) vs. the sum of the individual responses (3.8%, 32 of 843 cells) (Fig. 7d). Similar results were observed with ENaCδ siRNA (mixture: 4.5%, 38 of 849 cells responded, n = 17; sum: 4.4%, 37 of 849 cells responded; Fig. 7e). However, AR dramatically increased the number of NaCl-induced responses in NHE1 siRNA-transfected cells: responding cells increased from 5.1% (42 of 817 cells, n = 17) to 9.1% (74 of 817 cells) with the mixture (Fig. 7f). Apparently, the NHE1 receptor is not an essential element underlying the effect of AR on NaCl-elicited responses. These data are consistent with a model in which AR impacts salt responses via ENaC receptors, as indicated by our pharmacological and ENaCα and ENaCδ siRNA results. Furthermore, AR increased responses of amiloride-sensitive cells via interaction with ENaCα and ENaCδ receptors. Thus, both pharmacological and siRNA results show that AR increased responses of amiloride-sensitive cells via ENaCα and ENaCδ subunits.

**Figure 8:**
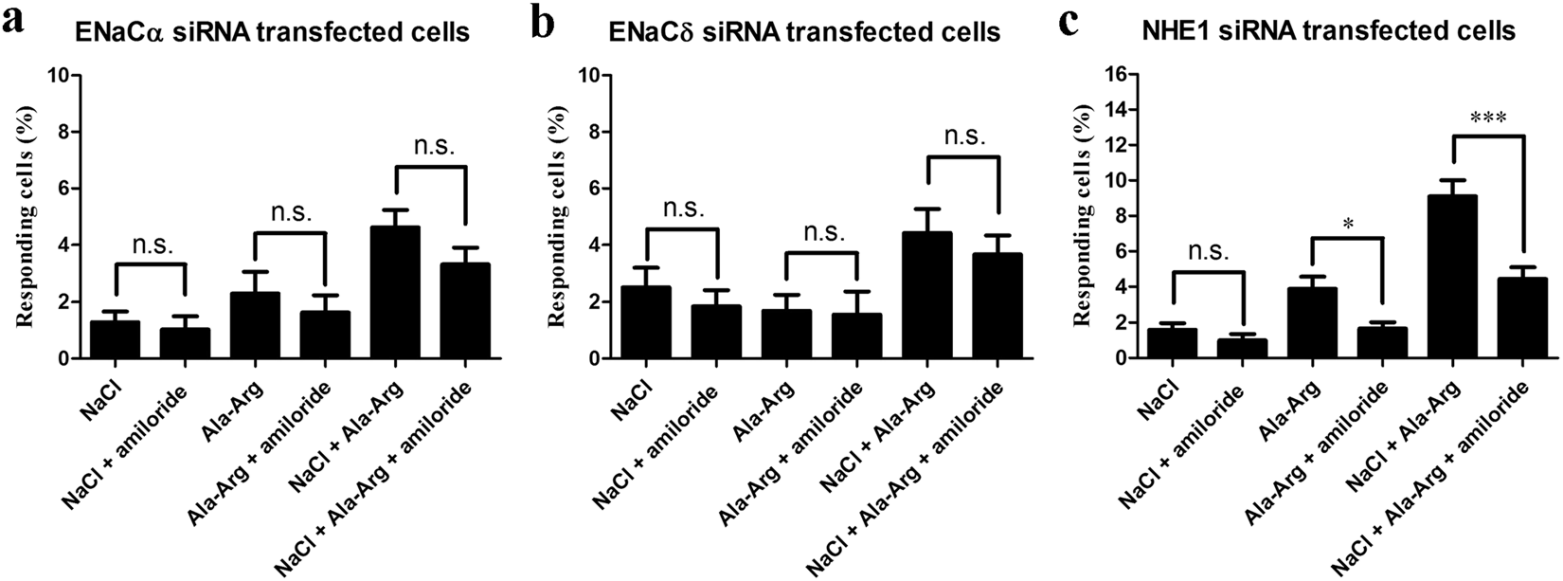
Effects of amiloride on NaCl (150 mM), Ala-Arg (AR; 50 μM), and the mixture of NaCl and AR in ENaCα (**a**), ENaCδ (**b**), and NHE1 (**c**) siRNA-transfected HBO cells. Responding cells were measured by intracellular Ca^2+^ changes. The concentration of amiloride was 50 μM. Each experiment was performed seventeen times. *p < 0.05, **p < 0.01, ***p < 0.001, n.s., not significant.

The pharmacological results indicated that another pathway might be involved in AR enhancement of cellular responses to NaCl. We next confirmed this hypothesis using ENaCα, ENaCδ, and NHE1 siRNA-transfected cells. No significant differences were observed between NaCl and the mixture of NaCl and amiloride, between AR and the mixture of AR and amiloride, or between the mixture of NaCl and AR and the mixture of NaCl, AR, and amiloride, in both ENaCα and ENaCδ siRNA-transfected cells (Fig. 8a,b). These results suggest that, in addition to amiloride-sensitive pathways, another pathway might be involved in the effect of AR on the responsiveness of these cells to NaCl. For NHE1 siRNA-transfected cells, no significant differences were observed between NaCl (1.6%, 13 of 817 cells, n = 17) and the mixture of NaCl and amiloride (1.0%, 8 of 817 cells; Fig. 8c). Additionally, amiloride significantly decreased AR-responding cells from 3.7% (11 of 294 cells, n = 6) to 1.7% (5 of 294 cells), and the mixture of NaCl and AR responses from 9.1% (74 of 817 cells, n = 17) to 4.4% (36 of 817 cells; Fig. 8c). These results suggest that AR did not influence the number of NaCl-elicited responses via the NHE1 receptor. These data are also consistent with a model in which AR acts via ENaC receptors to increase NaCl responses, as indicated by our pharmacological and ENaCα and ENaCδ siRNA results.

## Discussion

Although amino acids elicit taste on cellular, molecular, and behavioral levels^18, 21, 22^, few studies have focused on human sensory evaluation of the taste of dipeptides^19^. In our study, we observed that dipeptides elicited intracellular calcium changes in HBO cells at the micromolar level (Fig. 1a–c, Fig. S1a–d), suggesting that these dipeptides have taste. In general, amino acids and dipeptides elicit complex taste responses that cannot be described by a single characteristic^17, 20^. For example, L-alanine at low concentration (67 mM), primarily elicits sweet taste but both sweet and umami tastes at higher concentration (600 mM)^17^. Similarly, L-arginine at 5 mM mainly tastes bitter, but sweet taste can be detected at 10 mM^17^. The tastes of dipeptides are more complicated compared with single amino acids. There is no strict relationship in terms of taste between dipeptides and their constituent amino acids. At millimolar concentrations, arginyl dipeptides AR, RA, and RP elicit bitter taste, whereas RE and ER exhibit no taste at all; the non-arginyl dipeptides DD and ED elicit sour taste^20^.

No study has evaluated the enhancement of NaCl-induced responses by arginyl dipeptides. Using patented HBO cultured human fungiform taste papillae cells, we have demonstrated that three out of five arginyl dipeptides (AR, RA, and RP vs. RE and ER) increased the number of cells responding to NaCl, but not the non-arginyl dipeptides tested (DD and ED; Fig. 2a–f, Fig. S5a,b), indicating that the arginine residue is required but not sufficient to increase responses elicited by NaCl. Among AR, RA, and RP dipeptides, AR exhibited the strongest effect on responses to NaCl (Fig. 2a,d), yet no statistical difference was observed between AR and RA, indicating that the effect of amino acid order in these dipeptides is negligible. A previous report showed that arginyl dipeptides such as AR and RA at 12 mM enhanced the saltiness in 50 mM NaCl in human sensory evaluation^9^. Our results show that arginyl dipeptides, particularly AR and RA (50 µM), increased the number of HBO cells responding to NaCl (50 and 150 mM; Fig. 2a,b,d,e), consistent with the previous finding. Alanine (10, 50, and 100 µM), L-arginine (10, 50, and 100 µM), and their mixture (25 µM alanine + 25 µM arginine, and 50 µM alanine + 50 µM arginine) did not increase the number of HBO cell responses to NaCl (Fig. S4a,b), confirming that there is no strict relationship in terms of taste between dipeptides and their constituent amino acids. However, these results do not agree with the previous report that L-arginine (10 and 40 mM) enhanced amiloride-sensitive sodium membrane currents in αβγ-ENaC- or δβγ-ENaC-expressing oocytes and enhanced saltiness responses in subjects^23, 24^. The much higher concentrations (10 and 40 mM vs. 10, 50, and 100 µM, respectively) and use of different cell lines (αβγ-ENaC- or δβγ-ENaC-expressing oocytes vs. HBO cells) might explain the discrepancy.

Taste is a combination of modalities that functions by different mechanisms. Dipeptide AR (50 µM) elicited HBO cell responses that differed from those of sweet, bitter, umami, and salty stimuli (Fig. 3a–d), indicating that AR (50 µM) taste could not be described by these basic tastes. This characteristic resembles kokumi taste, which supplements, enhances, or modifies the original taste but does not have its own characteristic taste^25^.

Among the five proteins ENaC, NHE1, PLCβ2, LPAR1, and CaSR involved in taste response, NHE1 and PLCβ2 expressed in fungiform and circumvallate taste receptor cells are involved in the Na^+^ transport of common pathway for sweet, bitter, and umami taste transduction^26, 27^. LPAR1 initiates downstream signaling cascades through PLC, mitogen-activated protein kinase (MAPK), Akt, and Rho, which alter a range of cellular responses, including cell proliferation and survival, cell-cell contact through serum response element activation, cell migration and cytoskeletal changes, Ca^2+^ mobilization, and adenylyl cyclase inhibition^28^. CaSR is a class C G-protein-coupled receptor that is moderately activated by the aromatic amino acids (His, Trp, Phe, and Tyr) and weakly activated by Arg, Lys, Val, and Gly^29^. In addition, various extracellular CaSR agonists enhance sweet, salty, and umami tastes, whereas no taste exhibited by themselves^30, 31^. No cell response suppression was observed when HBO cells were presented with taste protein inhibitors cariporide, Ki16425, NPS2143, and U73122 (Fig. S5a–d). This suggests that AR-induced responses were not mediated by the interaction of AR with NHE1, LPAR1, CaSR, and PLCβ2. The ENaC inhibitor amiloride reduced the frequency of responses to AR (50 µM) alone and when mixed with NaCl (Fig. 4b,c), suggesting that ENaC is likely to be the main candidate receptor.

Taste bud cells utilize two principal salt transduction pathways: amiloride-sensitive and amiloride-insensitive^12^. ENaC is identified as an amiloride-sensitive receptor that is highly selective for sodium (and lithium) over other cations and particularly sensitive to low Na^+^ concentrations^13, 32^. Four homologous ENaC subunits, named α, β, γ, and δ, have been identified in human. Among them, the subunits α (or δ), β and γ, which are highly conserved in all vertebrates, are essential for the assembly of functional channels. The δ ENaC subunit exhibits maximal activity when co-expressed with β and γ subunits^33, 34^. Both pharmacological and siRNA studies indicated that AR increased NaCl-induced responses via the amiloride-sensitive pathways (Figs 4a–e, 7a–f). Further investigation of the ENaC subunits by siRNA showed that it is the ENaCα and ENaCδ subunits that are involved in the effect of AR on the number of NaCl-elicited responses (Fig. 7a–f). It has been reported that ENaC has two major states: open and closed. Hormones, proteases, ions, and signal transduction systems exert their regulatory role on ENaC activity by influencing directly or indirectly the probability of ENaC being in the open state, as well as the number of ENaC receptors in the apical membrane^33^. The mechanism by which AR affects the activity of ENaC remains to be investigated.

A variant of the transient receptor potential vanilloid-1 (TRPV1) has been proposed as the candidate amiloride-insensitive receptor, based on the observation that TRPV1-knockout mice had diminished taste nerve response of the amiloride-insensitive response to salty taste^5, 35, 36^, and amiloride-insensitive receptors are also proposed to be composed of at least two populations of taste cells sensing sour and bitter tastants^37^. However, currently, the receptor for amiloride-insensitive pathways remains unknown, and physiological studies indicate that this receptor responds to a broad range of organic and inorganic salts at relatively high concentrations. As amiloride inhibition and knockdown of ENaCα and ENaCδ subunits could not completely abolish cellular responses increased by AR and the mixture of AR and NaCl, we reason that in addition to amiloride-sensitive pathways, other pathways such as amiloride-insensitive pathways might be involved in AR increased cellular responses to NaCl (Fig. 8a–c). However, our pharmacological results also demonstrate that AR did not alter the responses of amiloride-insensitive cells (Fig. 4d). Therefore, we hypothesize that alternative dipeptide-related pathways might be involved in the increase in the frequency of cellular responses to NaCl by AR. Recently, proton-coupled oligopeptide transporter 1 (PEPT1), a member of the proton-coupled oligopeptide transporter (POT) family, was found to be responsible for di- or tripeptide transport in organisms^36^. In fact, almost all di- or tripeptides can be transported by PEPT1, which is a high-capacity, low-affinity transporter with affinities and inhibition constants ranging from 200 µM to 10 mM based on the substrate^36, 38, 39^. Whether PEPT1 is also a candidate receptor for the observed effect of AR on the number of cell responses to NaCl will need to be further investigated.

## Methods

### Solutions

NMDG Ringer solution, containing (in mM) NaCl 30, N-methyl-D-glucamine (NMDG) 115, KCl 5, CaCl_2_ 2, MgCl_2_ 1, 4-(2-hydroxyethyl)-1-piperazineethanesulfonic acid (HEPES) 20, and Na-pyruvate 1, was prepared along with Ringer solution, containing (in mM) NaCl 145, KCl 5, CaCl_2_ 2, MgCl_2_ 1, HEPES 10, and Na-pyruvate 1. Dipeptides AR, RA, RP, RE, ER, DD, and ED were synthesized using the solid-phase peptide synthesis technique and purified using reverse HPLC (NEOscientific) to achieve >98% purity and dissolved in Milli-Q water to prepare a stock of 100 mM. All amino acids used were L-isomers, and all dipeptides were composed of L-amino acids.

A sweet mixture comprising 1 mM sucralose and 100 mM glucose was used to ensure responses were from both artificial and natural sweet tastants. A bitter mixture comprising 2 mM phenylthiocarbamide, 5 mM salicin, and 2 mM denatonium benzoate was used because these bitters interact with different bitter receptors^40^. Monopotassium glutamate (MPG; 5 mM) was used for umami taste, and NaCl (150 mM) was used as salt stimuli. In the pharmacological studies, the specific blockers amiloride (50 µM), cariporide (5 µM), Ki16425 (0.2 µM), NPS2143 (0.5 µM), and U73122 (0.5 µM; Tocris Bioscience) were used. All the solutions were freshly prepared and adjusted to pH 7.0 and 300–310 mOsm/Kg·H_2_O before use.

### Criteria for selection of sample donors for fungiform papillae

Healthy, 22- to 40-year-old men (n = 2) and women (n = 2) were screened as potential donors of lingual fungiform papillae. Procedures were approved by an institutional review board (IRB) at the University of Pennsylvania and carried out in accordance with relevant guidelines and regulations. Subjects provided written, informed consent on IRB approved forms prior to testing. Subjects excluded on initial screening included those reporting a medical history or current manifestation of systemic, chronic disease; those reporting the regular use of prescription medications; and those reporting dry mouth or oral disease and the oral surgeon’s opinion that the subject was not appropriate for the study. The oral cavity was inspected for general tissue health. Subjects meeting these criteria were invited to participate in the study.

### Human fungiform taste papillae cells culture

Taste cells were cultured as previously reported^40–42^. Briefly, human fungiform taste papillae were removed from the anterior of the tongue. The papillae were incubated in isolation buffer mixed with soy trypsin inhibitor, collagenase, and elastase and then transferred to Iscove’s modified Dulbecco’s medium (Gibco) containing 10% fetal bovine serum, a 1:5 ratio of MCDB 153 (Molecular, Cellular, and Developmental Biology 153) medium (Sigma), 1% antibiotic-antimycotic, and 0.015% gentamycin (2.5 µg/mL). Cells with medium were gently resuspended, transferred to a sterile tissue culture flask, and incubated at 36 °C in a humidified incubator containing 5% CO_2_. One-third of the culture medium was replaced after 48 h and again after 5–7 days.

### Calcium imaging

Changes in intracellular calcium levels ([Ca^2+^]_i_) in response to stimuli were measured using calcium imaging^21, 22, 42^. Cultured HBO cells were seeded onto coverslips, grown for 3–5 days, and then loaded with 5 µM fura-2 AM (Santa Cruz, USA) and 10% Pluronic F127 (Life Technologies) dissolved in DMSO in NMDG Ringer solution for 1 h at 36 °C. The images were visualized with an inverted fluorescence microscope (Olympus) and a CCD camera (Photometrics). The stimulus delivery and removal were controlled by a two-channel peristaltic pump (Spetec). Cells were exposed to the stimulus for 1 min. The cells were provided with at least 5 min of recovery time between stimuli. Images were captured every 2 s during stimulus HBO applications, with excitation wavelengths of 340 and 380 nm and an emission wavelength centered at 510 nm. Cell focusing, defining regions, and image acquisition were controlled by Metafluor software (Molecular Devices).

For EC_50_ value determinations, six different concentrations (5, 10, 50, 100, 250, and 500 µM) of each dipeptide were tested. Each peptide was applied in random order on HBO cells in independent experiments and responses to peptide were measured by intracellular Ca^2+^ changes. Data from each concentration of peptide were collected from independent experiments. To evaluate dipeptide modulating NaCl-induced responses, cells were consecutively stimulated with three different stimuli in the following order: dipeptide alone, NaCl alone, and a mixture of NaCl and dipeptide. The concentrations of AR and RA were 10, 50, and 100 µM; the concentrations of other dipeptides were 50 and 100 µM. Both 50 and 150 mM NaCl were examined. To investigate the breadth of tuning of AR, HBO cells were consecutively stimulated in order with AR, sweet mixture, bitter mixture, and MPG. In pharmacological studies, cells were stimulated with three stimuli in order: a mixture of NaCl and AR, a mixture of NaCl, AR, and amiloride, and then the mixture of NaCl and AR again, to demonstrate that cells were as responsive as before amiloride exposure. To investigate whether AR increased responses of amiloride-insensitive cells, HBO cells received three stimuli in order: a mixture of NaCl and amiloride, a mixture of AR and amiloride, and a mixture of NaCl, AR, and amiloride. In siRNA studies, both siRNA-transfected and untransfected cells were stimulated by NaCl alone, AR alone, and a mixture of NaCl and AR. To evaluate whether other pathways were involved in the AR effect, siRNA-transfected cells were stimulated by the following six stimuli: NaCl alone; a mixture of NaCl and amiloride; AR alone; a mixture of AR and amiloride; a mixture of NaCl and AR; and a mixture of NaCl, AR, and amiloride.

### Transfection of siRNA

The ENaCα-, ENaCδ-, and NHE1-specific siRNAs (Qiagen FlexiTube Premix siRNA) were used to downregulate ENaCα, ENaCδ, and NHE1 expression, respectively. Scrambled siRNA (Qiagen) was used as a negative control. siRNAs were dissolved in RNase-free water following the manufacturer’s protocol. siRNA transfection was carried out as described previously^43^. Briefly, HBO cells were seeded onto coverslips in 12-well plates. After reaching 70–80% confluence, cells were transfected with 30 nM of prepared siRNA premix diluted in taste cell medium. The medium was changed 24 h post-transfection. Transfection efficiency was determined using qPCR and immunocytochemistry. Transfected cells were tested after 72 h by calcium imaging.

### Quantitative real-time RT-PCR

qPCR was conducted to determine knockdown efficiency^40^. HBO cells were transfected with siRNA and further incubated until 72 h post-transfection. Total RNA was extracted by a Purelink RNA Mini Kit (Life Technologies) following the manufacturer’s instructions. DNase treatment of RNA was conducted with the Turbo DNA-free kit (Life Technologies). RNA quality was measured using an Agilent RNA ScreenTape assay (Agilent Technologies). Total RNA was reversed transcribed into cDNA using high-capacity cDNA reverse transcription kits (Applied Biosystems). Relative quantitation of the expression of selected genes was performed using the QuantStudio^TM^12K real-time PCR system (Applied Biosystems) and ENaCα, ENaCδ, and NHE1 TaqMan gene expression assay kits (Applied Biosystems). PCR amplification was performed in a final volume of 20 µL containing 1 µL 20× TaqMan gene expression assay, 10 µL 2× TaqMan gene expression master mix, 4 µL cDNA, and 5 µL RNase-free water. The cycling conditions were 50 °C for 2 min, 95 °C for 10 min, followed by 40 cycles of 95 °C for 15 s, 60 °C for 1 min. Results were normalized using GAPDH (Applied Biosystems).

### Immunocytochemistry

Immunocytochemistry confirmed that siRNA transfection successfully downregulated ENaCα, ENaCδ, and NHE1 expression^42^. HBO cells were transfected with ENaCα, ENaCδ, and NHE1 siRNA, and further incubated for 72 h. Untransfected and scrambled siRNA-transfected cells were used as controls. The cells were fixed with 4% paraformaldehyde for 15 min at room temperature and then permeabilized with 10% methanol and 10% hydrogen peroxide in PBS for 15 min. Then cells were blocked with 3% goat serum, 3% bovine serum albumin in PBS for 1 h and stained with primary antibodies ENaCα and ENaCδ (LSBio) and NHE1 (Alomone Labs) overnight at 4 °C, followed by goat anti-rabbit secondary antibody (IgG Alexa 635, 1:500) for 30 min at room temperature. Coverslips were mounted using Vectashield with 4’,6-diamidino-2-phenylindole (DAPI; Vector Laboratories) after washing with PBS and water. Substituting the primary antibody with host IgG was used to determine the absence of nonspecific labeling. The photo was taken using a Leica TCS SP2 spectral confocal microscope (Leica Microsystems).

### Data analysis

The change in fluorescence ratio (*F*_340_/*F*_380_) was recorded for regions of interest (ROIs) were selected for each cell. Increases in intracellular Ca^2+^ evoked by stimulus application were expressed by Δ*F* = *F*_Peak _− *F*_Baseline_, where *F* = absorbance at 340/380 nm. The criterion for considering a responding cell was Δ*F* ≥ 0.03. Cells that failed to return to baseline were not counted, nor were apparent responses that occurred within 30 s or more than 120 s after stimulation. The percentage of responding cells was calculated by dividing the number of cells with a detectable Ca^2+^ increase by the total number of cells in the given experimental condition. The baseline fluorescence (Δ*F*/*F*) value of a cell was measured before taste stimuli given and the peak value of Ca^2+^ was measured after taste stimuli given. Statistical analysis of Ca^2+^ changes was conducted by counting the Ca^2+^ peaks during the first 2 min of stimulation. For each of the experiments, “n=” indicates number of experiment performed. Data were processed and plotted using Origin 8 (OriginLab) and Excel (Microsoft). Statistical comparisons between experiments were performed using a one-way analysis of variance and Tukey’s post-hoc test using GraphPad Prism (version 5.0; GraphPad Software). *p*-Values ≤ 0.05 were considered significant.

## Electronic supplementary material

Supplementary information
