# Supplementary material for: Arginyl dipeptides increase the frequency of NaCl-elicited responses via epithelial sodium channel alpha and delta subunits in cultured human fungiform taste papillae cells
Source: Sci Rep. 2017 Aug 8;7:7483. doi: 10.1038/s41598-017-07756-x (PMC5548727; doi:10.1038/s41598-017-07756-x)
Supplement: Supplementary file 1 — Supplementary information [file 41598_2017_7756_MOESM1_ESM.doc]

**Arginyl dipeptides increase the frequency of NaCl-elicited responses via epithelial sodium channel alpha and delta subunits in cultured human fungiform taste papilla cells**

Jiao-Jiao Xu^1^, Nadia Elkaddi^2^, Alvaro Garcia-Blanco^2^, Andrew I. Spielman^3^, Alexander A. Bachmanov^2^, Hau Yin Chung^1^*, Mehmet Hakan Ozdener^2^*


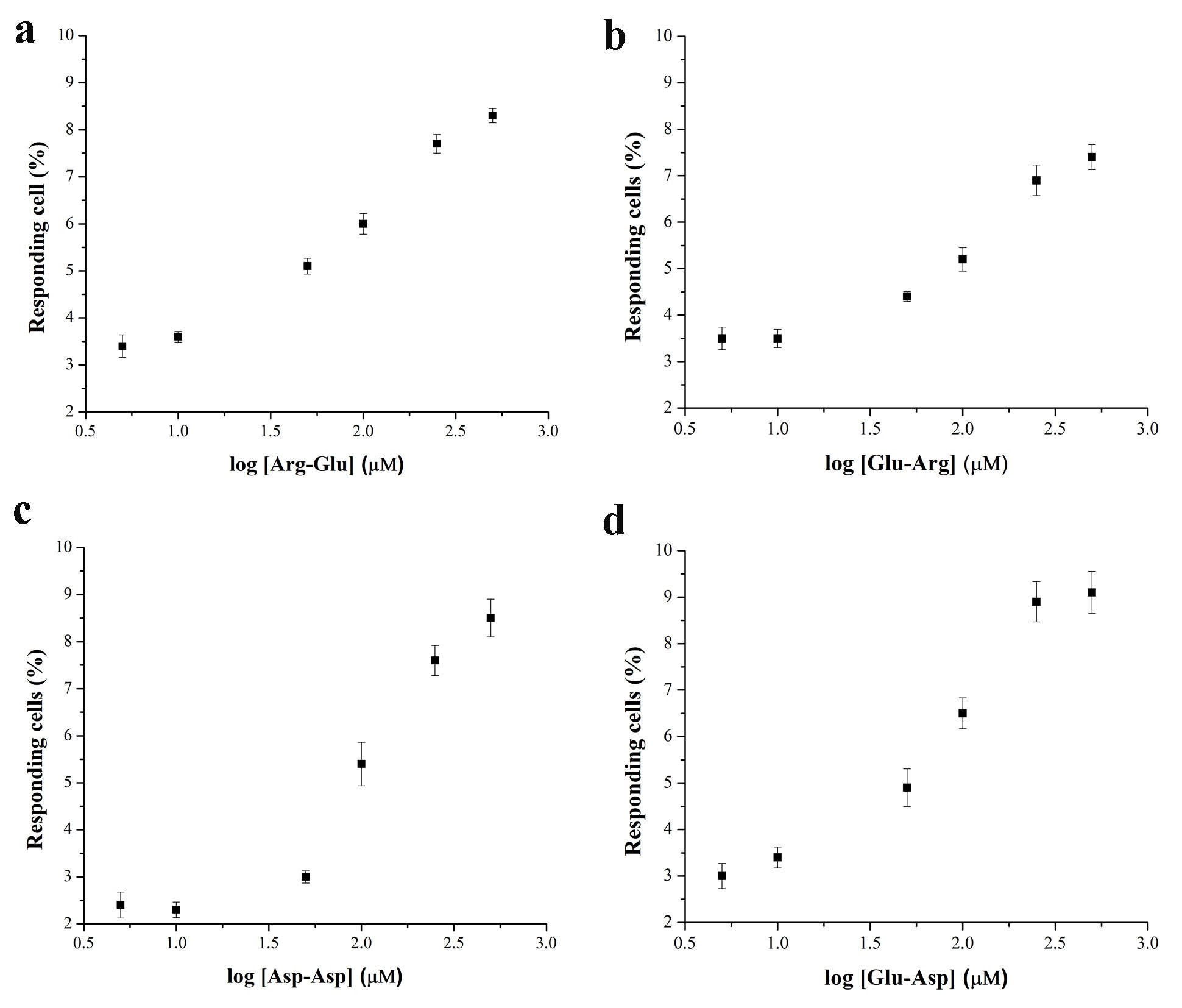


**Supplementary Figure 1. The concentration-response relationship for arginyl dipeptides Arg-Glu (a) and Glu-Arg (b) and nonarginyl dipeptides Asp-Asp (c), and Glu-Asp (c).** HBO cells were stimulated with six different dipeptide concentrations (µM: 5, 10, 50, 100, 250, and 500 (x-axis)) and each point represents the average of data collected from peptide-responding HBO cells. Dipeptides elicited responses in HBO cells in a concentration-dependent manner, as measured by intracellular Ca^2+^ changes. Each experiment was performed eight times. For each panel the peak number of responding cells/total cells examined were: 81/658 (a), 68/561 (b) and 60/424 (c), 107/556 (d).


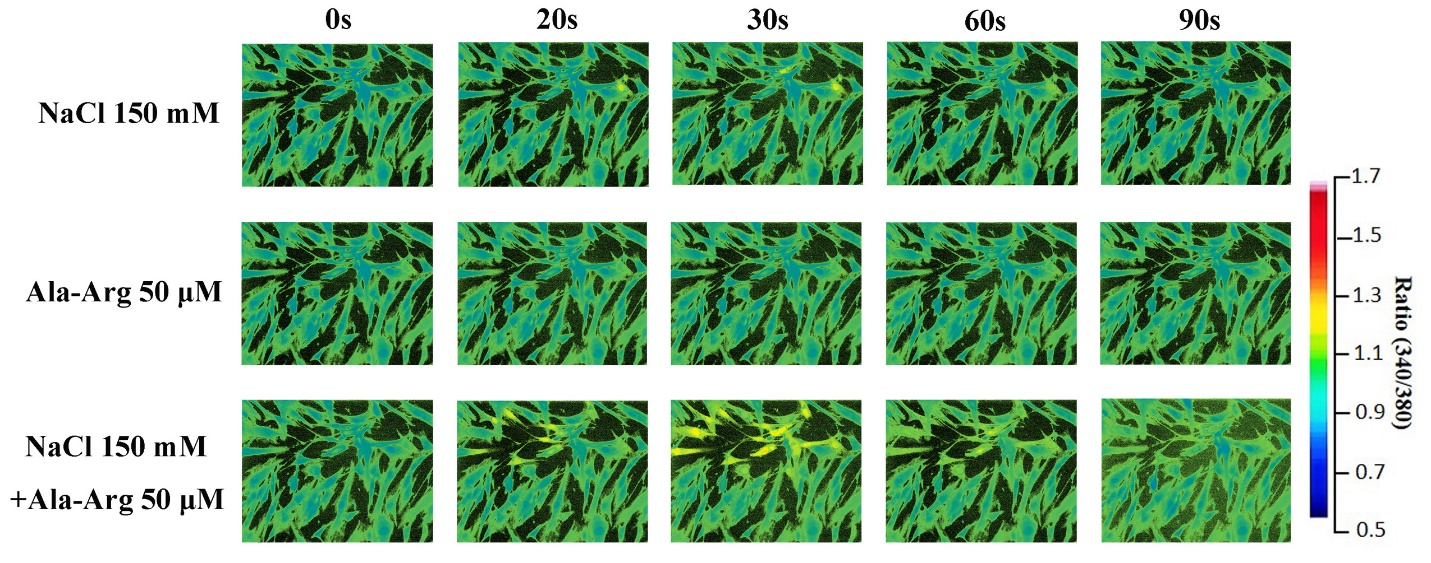


**Supplementary Figure 2. Ala-Arg (AR) dipeptides increase the frequency of NaCl-elicited responses.** Representative ratiometric images of Fura-2AM-loaded HBO cells. Ca2+ images of HBO cells were obtained at 0, 20, 30, 60, and 90 s after stimulus application. The color scale indicates the *F*_340_/*F*_380_ ratio. The Fura-2 ratio is encoded by pseudocolor.


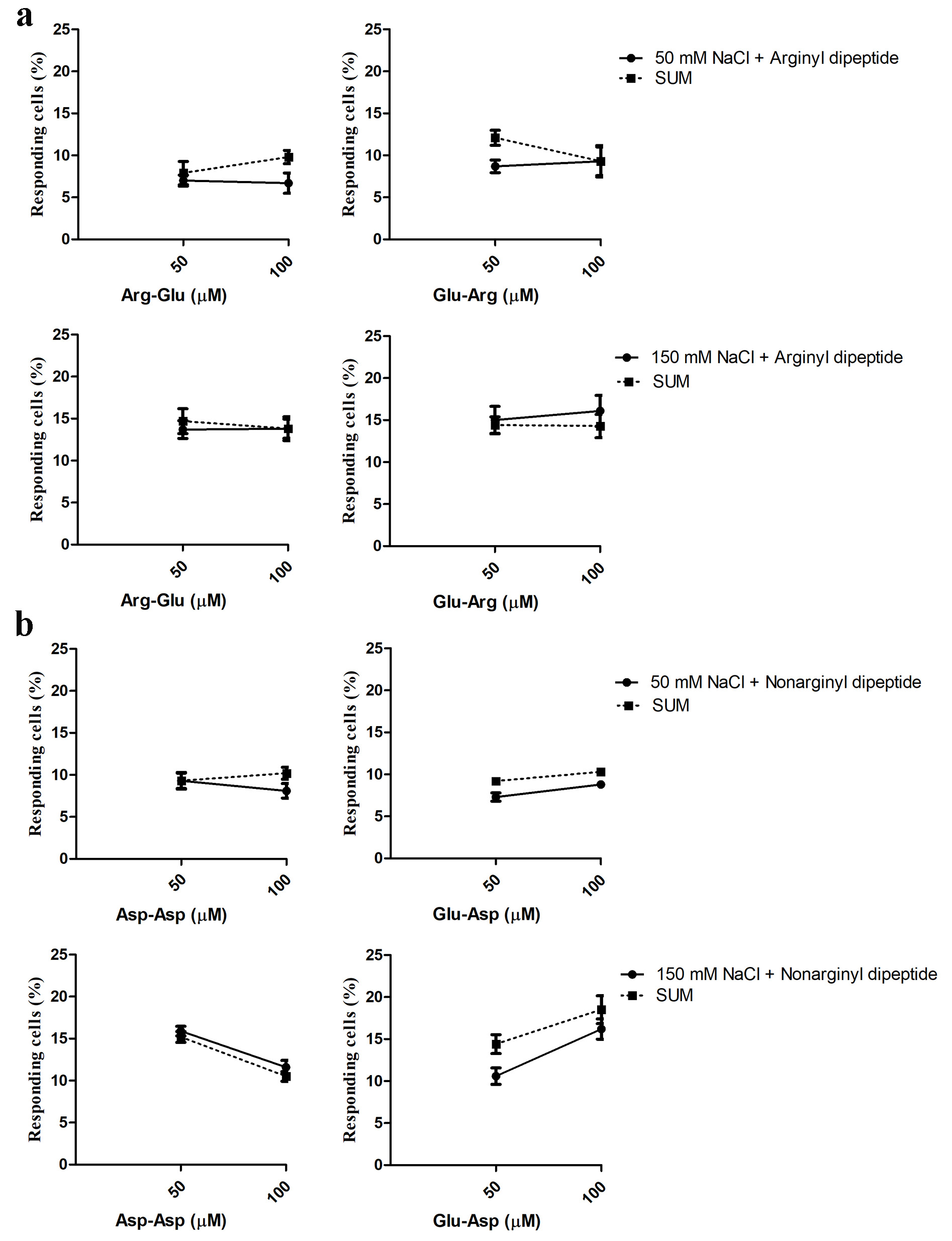


**Supplementary Figure 3. Effect of arginyl dipeptides (a) and nonarginyl dipeptides (b) on the number of NaCl-elicited responses.** HBO cells were consecutively stimulated by three different stimuli: NaCl alone (two different concentrations: 50 and 150 mM), dipeptide alone (50 or 150 µM), and the mixture of NaCl and dipeptide. SUM is the mathematical addition of individual responses (NaCl alone + dipeptide alone). Results compared the percentage of responding cells between the mixture and SUM. The x-axis represents dipeptide concentrations (µM). Y-axis indicates responding cells measured by intracellular Ca^2+^ changes in HBO cells. Each experiment was performed in six duplicates (n=6). No significant differences were observed between the mixture and the SUM. For each panel the peak number of responding cells/total cells examined were: (a) at 50 mM NaCl 19/452 for RE and 19/347 for ER, at 150 mM NaCl 52/546 for RE and 76/718 for ER, (b) at 50 mM NaCl 15/369 for DD and 18/422 for ED, at 150 mM NaCl 66/686 for DD and 50/458 for ED.


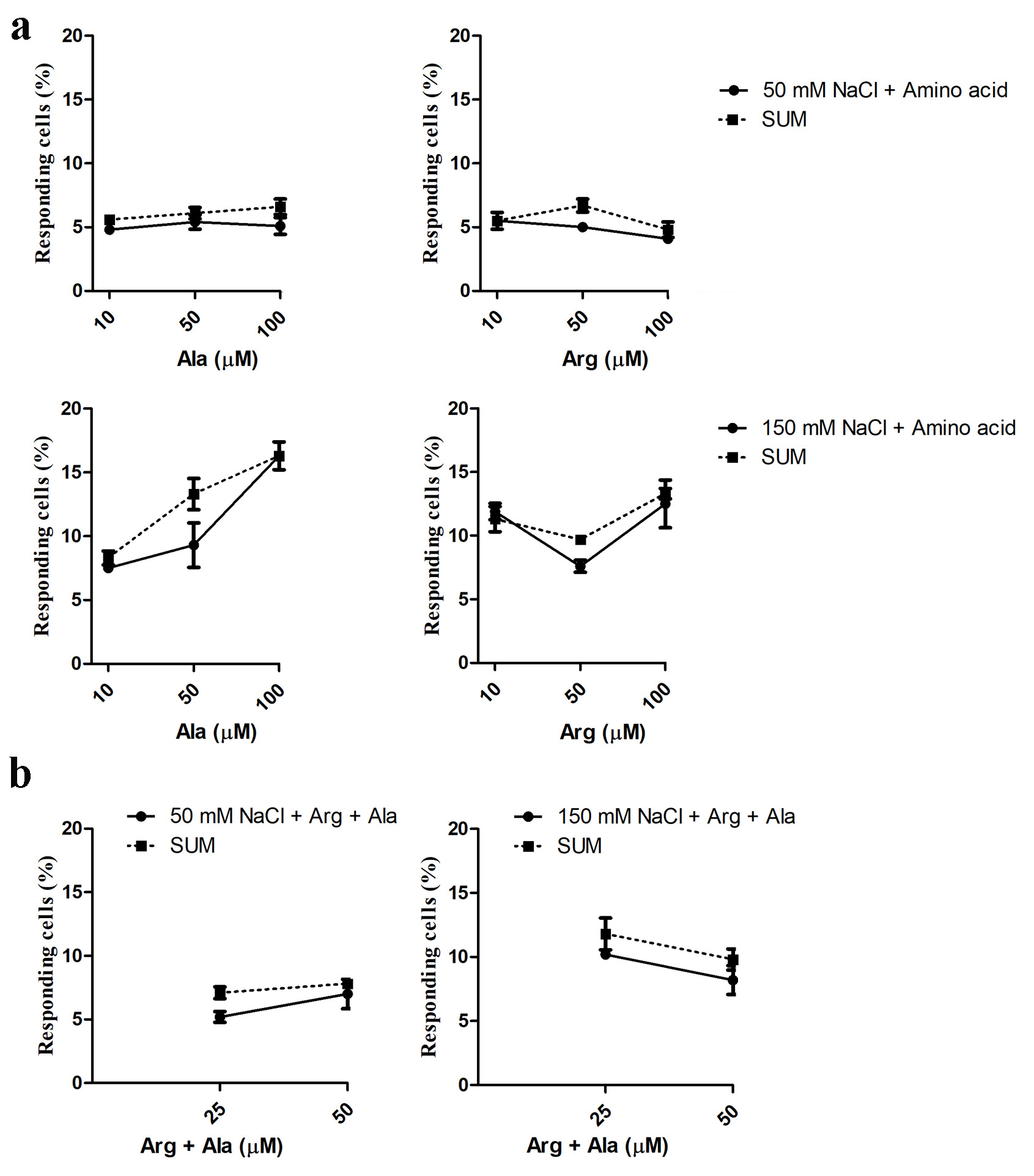


**Supplementary Figure 4. Effects of alanine (Ala), arginine (Arg) (a), and their mixture (b) on the number of NaCl-responding cells.** HBO cells were consecutively stimulated by the following stimuli: NaCl alone (50 and 150 mM), amino acids alone, and the mixture of NaCl and amino acid(s). The percentage of cells responding to the stimuli were measured by intracellular Ca^2+^ changes. Each experiment was performed five times. SUM is the mathematical addition of individual responses (NaCl alone and amino acids alone). No significant differences were observed between the mixture and the SUM. For each panel the peak number of responding cells/total cells examined were: (a) at 50 mM NaCl 20/411 for A and 21/472 for R, at 150 mM NaCl 59/418 for A and 42/424 for R, (b) at 50 mM NaCl 14/282 for R+A and at 150 mM NaCl 24/311 for R+A.


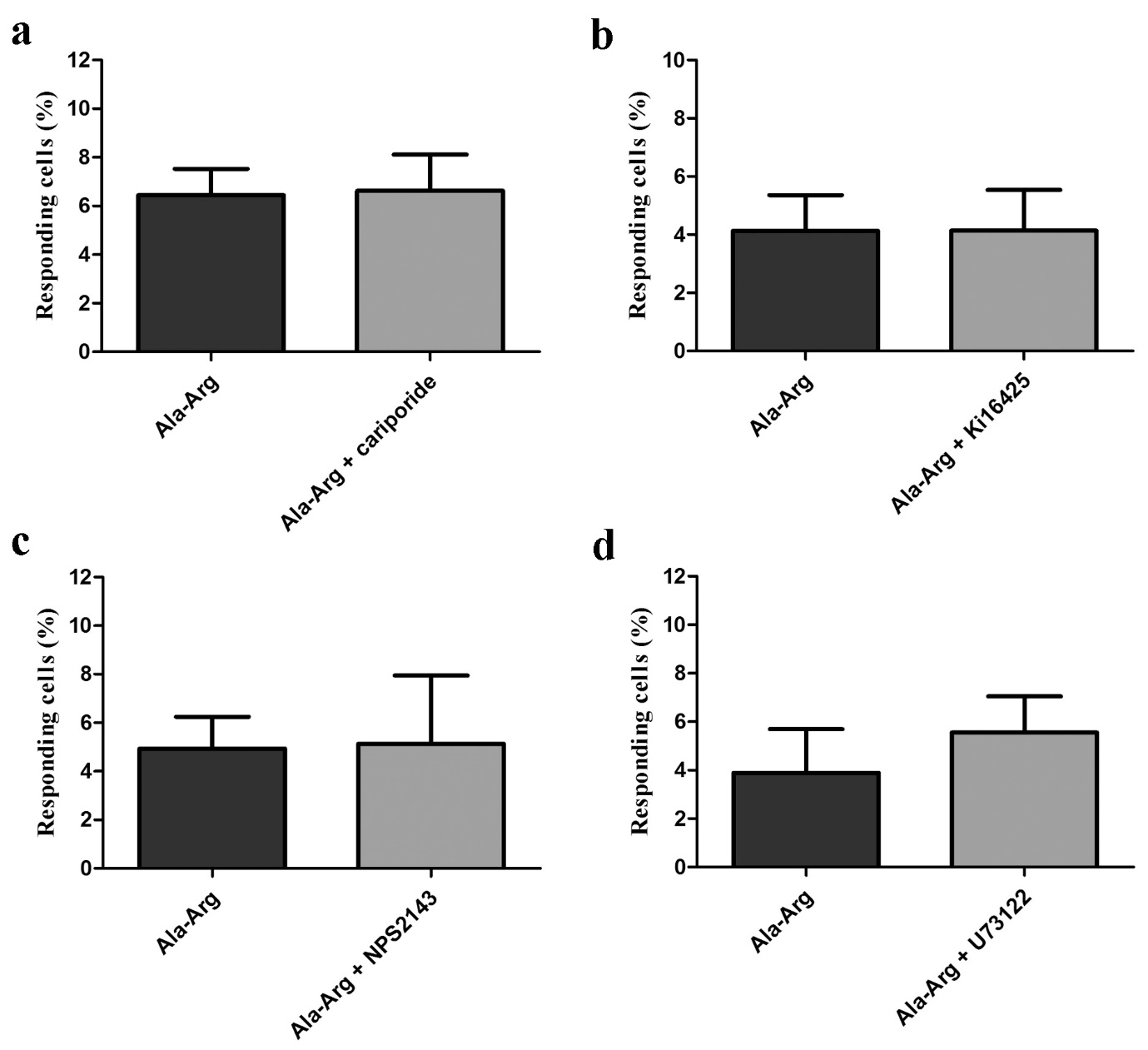


**Supplementary Figure 5. Effects of blockers cariporide (5 µM), Ki16425 (0.2 µM), NPS2143 (0.5 µM), and U73122 (0.5 µM) on Ala-Arg (AR)-elicited responses.** HBO cells were stimulated by two stimuli: AR alone and a mixture of AR and blockers. Responses elicited by AR were not inhibited by cariporide **(a)**, Ki16425 **(b)**, NPS2143 **(c)**, or U73122 **(d)**. Cell responses were measured by intracellular Ca^2+^ changes. Each experiment was performed three times. No significant differences were observed.
